# Comparing the normalization methods for the differential analysis of Illumina high-throughput RNA-Seq data

**DOI:** 10.1186/s12859-015-0778-7

**Published:** 2015-10-28

**Authors:** Peipei Li, Yongjun Piao, Ho Sun Shon, Keun Ho Ryu

**Affiliations:** College of Electrical and Computer Engineering, Chungbuk National University, Cheongju-si, South Korea

**Keywords:** RNA-Seq data, Gene expression, Normalization, Correlation, Poly-A tails

## Abstract

**Background:**

Recently, rapid improvements in technology and decrease in sequencing costs have made RNA-Seq a widely used technique to quantify gene expression levels. Various normalization approaches have been proposed, owing to the importance of normalization in the analysis of RNA-Seq data. A comparison of recently proposed normalization methods is required to generate suitable guidelines for the selection of the most appropriate approach for future experiments.

**Results:**

In this paper, we compared eight non-abundance (RC, UQ, Med, TMM, DESeq, Q, RPKM, and ERPKM) and two abundance estimation normalization methods (RSEM and Sailfish). The experiments were based on real Illumina high-throughput RNA-Seq of 35- and 76-nucleotide sequences produced in the MAQC project and simulation reads. Reads were mapped with human genome obtained from UCSC Genome Browser Database. For precise evaluation, we investigated Spearman correlation between the normalization results from RNA-Seq and MAQC qRT-PCR values for 996 genes. Based on this work, we showed that out of the eight non-abundance estimation normalization methods, RC, UQ, Med, TMM, DESeq, and Q gave similar normalization results for all data sets. For RNA-Seq of a 35-nucleotide sequence, RPKM showed the highest correlation results, but for RNA-Seq of a 76-nucleotide sequence, least correlation was observed than the other methods. ERPKM did not improve results than RPKM. Between two abundance estimation normalization methods, for RNA-Seq of a 35-nucleotide sequence, higher correlation was obtained with Sailfish than that with RSEM, which was better than without using abundance estimation methods. However, for RNA-Seq of a 76-nucleotide sequence, the results achieved by RSEM were similar to without applying abundance estimation methods, and were much better than with Sailfish. Furthermore, we found that adding a poly-A tail increased alignment numbers, but did not improve normalization results.

**Conclusion:**

Spearman correlation analysis revealed that RC, UQ, Med, TMM, DESeq, and Q did not noticeably improve gene expression normalization, regardless of read length. Other normalization methods were more efficient when alignment accuracy was low; Sailfish with RPKM gave the best normalization results. When alignment accuracy was high, RC was sufficient for gene expression calculation. And we suggest ignoring poly-A tail during differential gene expression analysis.

**Electronic supplementary material:**

The online version of this article (doi:10.1186/s12859-015-0778-7) contains supplementary material, which is available to authorized users.

## Background

Bioinformatics studies over the past few decades have shed light on topics such as sequence analysis, structural analysis, and network and systems biology [1, 2]. In recent years, rapid improvements in technology and decreased sequencing costs have made next-generation sequencing possible, facilitating millions of short sequence reads that have broad genomic research applications [3–5]. Using next-generation sequencing to study the RNA in a sample (i.e., RNA-Seq) allows for whole transcriptome shotgun sequencing, which is useful in analyses such as gene expression analysis [6, 7], single nucleotide variation discovery [8], and fusion gene detection [9, 10]. In our work, we focused on accurately quantifying gene expression levels using deep-sequencing methods. An advantage of RNA-Seq is that it overcomes many of the limitations of previous microarray technologies, such as the dependence on prior knowledge of hybridization, limitations to measurement accuracy and, particularly, low sensitivity for transcript detection [11–13]. However, the estimation of mRNA abundance from RNA-Seq data is not a simple task because the sequence data produced are numerous and complicated, and cannot be directly interpreted. Therefore, similar to other high-throughput technologies, the analysis methodology is critical for accurate data interpretation. Even though various technologies have been proposed in recent years for RNA-Seq analysis, it is a technology that is still being actively developed.

In general, the RNA-Seq pipeline for differential expression analysis contains five steps [14, 15]. First, long RNA samples are fragmented into short complementary DNA (cDNA) fragments, and are then sequenced on a high-throughput platform such as Illumina. The resulting short sequence reads are then mapped back to the reference genome or transcriptome. After that, the gene expression level is estimated for each gene or isoform. The summarized data are then normalized using statistical approaches or machine learning algorithms to identify differentially expressed genes. Finally, the significance of the data is determined in a biological context.

As with microarrays [16, 17], various artifacts and biases affect quantification results. Therefore, normalization is an essential step in analyzing differential expression of genes from RNA-Seq data [18]. Many non-abundance estimation normalization methods have been proposed to correct biases between and within samples. Raw count (RC), upper quartile (UQ), and median (Med) make up the general descriptive statistical methods that are widely used in capturing data characteristics. Other inter-sample normalization methods calculate scale factors according to library size, which is the total number of mapped reads. Trimmed mean of M-values (TMM) normalization is a simple and effective method for estimating relative RNA production levels from RNA-Seq data [19]. The TMM method estimates scale factors between samples, and can be incorporated into currently used statistical methods for differential expression analysis. DESeq is based on negative binomial distribution, with variance and mean linked by local regression, and presents an implementation that also gives scale factors [6]. Quantile (Q) was first proposed in the context of microarray data; it is a normalization method that involves matching distributions of gene counts across runs [20]. Reads per kilobase per million mapped reads (RPKM) is the most widely used method in next-generation sequencing research for length normalization. This approach was initially introduced to facilitate comparisons between genes within a sample, and combines inter- and intra-sample normalization, because it rescales gene counts to correct for differences in both library size and gene length [21]. ERPKM is an improvement on RPKM because it uses an effective read length.

In addition to previously introduced traditional normalization methods, two abundance estimation normalization methods have recently been developed for accurate estimation: RNA-Seq by Expectation-Maximization (RSEM) [22] and Sailfish [23]. These methods are completely different from the previously proposed methods because they use machine learning algorithms to conduct abundance estimation. For example, RSEM proposes a statistically directed graph model and uses the expectation–maximization algorithm to estimate abundances at the gene level considering multiple variables derived from RNA-Seq and transcript data, including library sizes and gene lengths. Sailfish is another new approach for quantifying abundance; it avoids mapping reads to reference transcripts and uses counts of k-mers to estimate transcript coverage. This method makes an innovative step by eliminating the need for alignment, and may save a great deal of time in the matching step.

Among the various normalization methods, the one that produces the best results must be determined. A comparison of the recently proposed normalization methods will provide clearer guidelines for future analyses. In a previous study [24], several methods were compared and useful suggestions made; however, comparisons were not made with the recent abundance estimation approaches, which have proven to be quite efficient.

In this study, for gene expression analysis, we first compared eight non-abundance estimation normalization methods (RC, UQ, Med, TMM, DESeq, Q, RPKM, and ERPKM) and then compared two abundance estimation normalization methods (RSEM and Sailfish) by combining them with the non-abundance methods. The experiments are based on real Illumina high-throughput RNA-Seq data used in the MicroArray Quality Control (MAQC) project from two RNA samples of brain tissue and a mixture of tissue types, with read lengths of 35 and 76 nucleotides. Simulation data were also obtained using a model derived from RNA-Seq data with a sequence length of 76 nucleotides by RSEM simulator. Reads are aligned with human genome data obtained from the University of California Santa Cruz (UCSC) Genome Browser Database. First, large-scale distribution and detail alignment results were represented. Then, we analyzed Spearman correlation coefficients between the normalization results from RNA-Seq and the results of MAQC TaqMan quantitative reverse transcription polymerase chain reaction (qRT-PCR) of two samples for 996 genes. We have provided a detailed comparison of results among the different normalization methods. In addition, the impact of the poly-A tail was verified by adding 0, 5, 10, 15, 20, or 25 adenine bases to the end of the transcript data. Based on this study, we propose practical recommendations on the appropriate normalization method to use, and determine the effect of adding a poly-A tail to gene expression analyses.

## Results and discussion

### Alignment

For real reads of 35 nucleotides, the range of alignment counts was from 0 to approximately 9,000,000. For approximately 70 % of the genes, the alignment count was between 1000 and 100,000. Only 14 genes had a count number greater than 1,000,000. In one respect, this proves that only a small number of genes can be differentially expressed. For each run, the total number of reads was approximately 11,000,000–16,000,000.

The number of reads with at least one alignment (with no poly-A tail) was approximately 500,000–700,000 (about 5 % of the total reads), and the number with total alignment was approximately 1,500,000–5,000,000.

For real reads of 76 nucleotides, the number of reads with at least one alignment (with no poly-A tail) was approximately 300,000–500,000 (about 3 % of the total reads). The number of alignments decreased as read length increased. This proves that longer reads give more accurate alignment results.

The low rate of alignment arose because we only used 996 genes. If we had used full human genes as references, this would have been very different. For example, on real reads of 35 nucleotides, the alignment rate was approximately 55 %, and on real reads of 76 nucleotides, the alignment rate was around 65 %.

Detailed alignment results for each accession can be found in Additional file 1.

After alignment we removed reference transcripts with no mappings for each accession.

### Comparison of normalization methods

We investigated Spearman correlation coefficients between normalization results from RNA-Seq using each normalization method and the values from qRT-PCR.

Table 1 gives the Spearman correlation coefficient results for eight non-abundance estimation normalization methods (not applying abundance estimation normalization). Table 2 gives Spearman correlation coefficient results for two abundance estimation methods (RSEM and Sailfish) combined with RC and RPKM.

**Table 1:** Spearman correlation results of eight non-abundance estimation normalization methods

Accession	RC	UQ	Med	TMM	DESeq	Q	RPKM	ERPKM
Reads with length of 35 nucleotides, *p*-value < 2.2e-16								
SRX016359	0.563	0.561	0.563	0.563	0.563	0.563	0.560	0.560
SRX016366	0.562	0.560	0.563	0.563	0.563	0.562	0.559	0.559
SRX016367	0.622	0.621	0.622	0.622	0.622	0.622	0.639	0.639
SRX016368	0.621	0.620	0.622	0.621	0.621	0.621	0.639	0.639
SRX016369	0.626	0.625	0.626	0.626	0.626	0.626	0.646	0.646
SRX016370	0.635	0.635	0.635	0.635	0.635	0.635	0.657	0.657
SRX016371	0.632	0.631	0.632	0.632	0.630	0.632	0.652	0.651
SRX016372	0.641	0.641	0.641	0.6401	0.641	0.640	0.662	0.662
Reads with length of 76 nucleotides, *p*-value < 2.2e-16								
SRX080222	0.695						0.653	0.650
SRX080223	0.686						0.642	0.640
SRX080224	0.713						0.695	0.693
SRX080225	0.712						0.693	0.692
Simulated-HBR	0.670	0.669	0.670	0.670	0.670	0.670	0.624	0.621
Simulated-UHR	0.708	0.708	0.707	0.708	0.708	0.708	0.685	0.683

**Table 2:** Spearman correlation results of two abundance estimation methods combined with RC and RPKM

Accession	RC	RPKM	RSEM + RC	RSEM + RPKM	Sailfish + RC	Sailfish + RPKM
Reads with length of 35 nucleotides, *p*-value < 2.2e-16						
SRX016359	0.563	0.560	0.690	0.692	0.696	0.694
SRX016366	0.562	0.559	0.689	0.691	0.700	0.695
SRX016367	0.622	0.639	0.755	0.778	0.752	0.797
SRX016368	0.621	0.639	0.755	0.777	0.752	0.797
SRX016369	0.626	0.646	0.770	0.794	0.748	0.806
SRX016370	0.635	0.657	0.778	0.802	0.766	0.815
SRX016371	0.632	0.652	0.773	0.795	0.760	0.811
SRX016372	0.641	0.662	0.781	0.804	0.772	0.819
Reads with length of 76 nucleotides, *p*-value < 2.2e-16						
SRX080222	0.695	0.653	0.691	0.650	0.570	0.583
SRX080223	0.686	0.642	0.682	0.639	0.555	0.575
SRX080224	0.713	0.695	0.711	0.693	0.535	0.602
SRX080225	0.712	0.693	0.709	0.690	0.530	0.597
Simulated-HBR	0.670	0.624	0.667	0.622	0.557	0.579
Simulated-UHR	0.708	0.685	0.705	0.683	0.558	0.629

All the Spearman correlation coefficient results were larger than 0, indicating that the results from all the RNA-Seq normalization methods were positively correlated with the qRT-PCR values. Furthermore, all p-values were < 2.2e-16 and the null hypothesis was rejected, which proves that all the results of the normalization methods correlated with the qRT-PCR gene expression values. In another aspect, the RNA-Seq data were compatible with the real time PCR for gene expression analysis.

As can be seen in Table 1, the Spearman correlation coefficients for RC, Med, TMM, and DESeq were all 0.563 for accession SRX016359, and for Med, TMM, and DESeq they were 0.563 for SRX016366. For other accessions, RPKM and ERPKM generated results that correlated more than those from the other methods; RPKM gave correlations of 0.639, 0.639, 0.646, 0.657, 0.652, and 0.662 for accessions SRX016367, SRX016368, SRX016369, SRX016370, SRX016371, and SRX016372, respectively. This proves that consideration of the transcript length in normalization is quite effective. However for SRX080222, SRX080223, SRX080224, and SRX080225, RC achieved better correlation than RPKM and ERPKM. Moreover, simulated data correlations did not increase after normalization methods were applied, and for RPKM and ERPKM correlation actually decreased. For all accessions, ERPKM did not achieve better results than RPKM. Using an effective transcript length obviously does not improve normalization results.

Table 2 reveals that for all eight accessions with a read length of 35 nucleotides, Sailfish with RPKM achieved the best Spearman correlation coefficient results, followed by RSEM, which also improved a great deal on the results for RC when no abundance-estimation normalization method was used. For accession SRX016372, a highest correlation of 0.819 was achieved, which shows that RNA-Seq can precisely predict gene expression levels. In practice, Sailfish with RPKM could almost replace qRT-PCR measurements. However, for sequence data on 76-nucleotide sequences, RC with no abundance-estimation normalization method applied achieved the best results, followed by RSEM with similar correlations; Sailfish generated much worse correlation values.

From the comparison results shown above, normalization methods are not necessary for all sequence data. Inter-sample normalization methods, such as TMM, DESeq, and Q, which scale sample size, do not noticeably improve gene expression, regardless of read length. However, RPKM is likely to be more efficient when alignment accuracy is low. Similarly, for read data on lengths of 35 nucleotides, of the two abundance estimation normalization methods, Sailfish with RPKM, which is also quite an efficient combination because it is alignment-free, gave better normalization results than RSEM. However, when alignment accuracy is high, RC seems to be adequate for gene expression calculations in real experiments.

For all details of the Spearman correlation coefficient results, please download Additional file 2.

### Comparison of poly-A tails

To determine whether a poly-A tail can affect the alignment results, we evaluated the results of accession SRX016359 with the addition of various poly-A tail lengths to the end of the reference transcript data. Because the read length was 34 nucleotides, we used poly-A tail lengths of 0, 5, 10, 15, 20, and 25 adenines. Table 3 shows the alignment count numbers for each run. When the length of the poly-A tail was increased, the alignments were also increased by a small number (relative to the total number). In other words, more reads can be mapped to a reference transcript by increasing the poly-A tail length.

**Table 3:** Total alignment numbers with different poly-A tail lengths on run SRX016359

Accession	Runs	0A	5A	10A	15A	20A	25A
SRX016359	SRR035678	4,373,013	4,373,128	4,373,220	4,373,338	4,374,306	4,840,614
	SRR037439	2,028,240	2,028,376	2,028,503	2,028,622	2,028,900	2,069,175
	SRR037440	4,385,598	4,385,761	4,385,880	4,386,034	4,386,793	4,794,397
	SRR037441	2,161,858	2,162,012	2,162,156	2,162,303	2,162,646	2,205,932
	SRR037442	4,840,368	4,840,534	4,840,669	4,840,802	4,841,777	5,319,375
	SRR037443	2,043,229	2,043,370	2,043,531	2,043,665	2,043,916	2,070,547
	SRR037444	1,939,846	1,939,970	1,940,096	1,940,204	1,940,597	1,988,929

Table 4 shows Spearman correlation coefficient results for the eight non-abundance estimation normalization methods with different poly-A tails. Correlations were either unchanged or minimally decreased when comparing 0 adenines with 5, 10, 15, 20, and 25 adenines. The data show that by adding a short poly-A tail, few relative alignments are necessary but, compared with the total alignment number, the number was so small that normalization results could not be improved. Adding poly-A tails that were too long caused irrelevant alignments to be included, which negatively impacted the normalization results. In summary, choosing appropriate poly-A tail lengths may improve differential analysis; however, based on the minimal effect observed in this study, poly-A tail length can probably be ignored.

**Table 4:** Spearman correlation results of eight non-abundance estimation normalization methods by adding a poly-A tail

Accession	RC	UQ	Med	TMM	DESeq	Q	RPKM	ERPKM
SRX016359-0A	0.563	0.561	0.563	0.563	0.563	0.563	0.560	0.560
SRX016359-5A	0.563	0.561	0.563	0.563	0.563	0.563	0.560	0.560
SRX016359-10A	0.563	0.561	0.563	0.563	0.563	0.563	0.560	0.560
SRX016359-15A	0.563	0.561	0.563	0.563	0.563	0.563	0.560	0.560
SRX016359-20A	0.563	0.560	0.563	0.563	0.563	0.562	0.560	0.559
SRX016359-25A	0.544	0.536	0.551	0.544	0.543	0.544	0.537	0.536

## Conclusions

Normalization has proved to be important in the analysis of gene expression using RNA-Seq technology. Recently, various normalization approaches have been developed to accurately identify differentially expressed genes. To provide a guideline for choosing among these methods, we compared eight non-abundance estimation normalization methods (RC, UQ, Med, TMM, DESeq, Q, RPKM, and ERPKM) and two abundance estimation normalization methods (RSEM and Sailfish) in this study. The experiments were based on the real Illumina high-throughput RNA-Seq data used in the MAQC project on two RNA samples, brain tissue (HBR) and a mixture of tissue types (UHR), with read lengths of 35 and 76 nucleotides. Simulated data were obtained using an RSEM simulator with parameters derived from real data with a length of 76 nucleotides. Reads were mapped with human genome data using the Bowtie tool. First, we showed a large-scale and detailed distribution of all alignments. We then investigated the Spearman correlation coefficient between the normalization results of each method and the values of MAQC TaqMan qRT-PCR. For accessions with a read length of 35 nucleotides, of the eight non-abundance estimation normalization approaches, RPKM achieved a higher correlation value than RC, UQ, Med, TMM, or Q, proving that consideration of the transcript length in normalization is quite effective. By using effective transcript lengths, ERPKM did not improve the normalization results compared with RPKM. After combining the abundance estimation normalization methods, the normalization results were improved. In particular, Sailfish with RPKM, which we recommend researchers use as a normalization method in future analyses, can almost replace qRT-PCR, as a correlation of nearly 0.8 was observed. Moreover, Sailfish is alignment-free and more time-efficient than RSEM. RSEM also produced good results. However, for data with read lengths of 76 nucleotides, none of the normalization methods improved the correlation results. Therefore, we conclude that when alignment accuracy is high, RC is sufficient for gene expression calculation in real experiments. In addition, the impact of poly-A tail was determined by adding adenines (0–25) to the transcript data. The results showed that by adding short poly-A tails, few relative alignments were required, but longer poly-A tails caused irrelevant alignments to be included. However, our results were not improved. Thus, choosing appropriate poly-A tail lengths may improve differential analysis, but did not appear to have an impact in this study. Therefore, we suggest that researchers do not need to consider ploy-A tails in the normalization step in gene differential expression analysis.

## Methods

### Reference transcript data

Human genome data (hg19, GRCh37) were obtained from the UCSC Genome Browser Database (http://genome.ucsc.edu/) for use as reference transcript data [25]. These data were first pre-processed at the gene level. The authors of previous studies have reported that reads extending into poly-A tails are challenging to align at the genome level [22, 26]. To determine whether a poly-A tail does or does not affect normalization results, we added different poly-A tails (0, 5, 10, 15, 20, and 25 adenines) to the reference transcript data for accurate comparison.

### Real RNA-Seq data

High-throughput RNA-Seq data were collected from the Sequence Read Archive (SRA; http://www.ncbi.nlm.nih.gov/sra) [27]. Data with a read length of 35 nucleotides were obtained from accession number SRA010153.1. Data with a read length of 76 nucleotides were obtained from accession number SRA039286. Raw data were generated from samples used in the MAQC project on two RNA samples, brain tissue (HBR) and a mixture of tissue types (UHR), through an Illumina genome analyzer.

As shown in Table 5, two types of biological samples (HBR and UHR) were assayed in MAQC-2, each using seven lanes distributed across two flow cells. In MAQC-3, four different UHR library preparations were assayed using 14 lanes from two flow cells, and each library preparation was assayed on only one of the flow cells. For accession number SRA039286, two types of biological sample (HBR and UHR) were used, and each had just a single run.

**Table 5:** RNA-Seq data description

Accession	Description	Sample	Read length	Runs	Size
SRX016359	MAQC Brain exp 2 using phi X control lane	HBR	35 nucleotides	7	3 Gb
SRX016366	MAQC Brain exp 2 using auto calibration	HBR	35 nucleotides	7	3 Gb
SRX016367	MAQC UHR exp 2 using phi X control lane	UHR	35 nucleotides	7	3.4 Gb
SRX016368	MAQC UHR exp 2 using auto calibration	UHR	35 nucleotides	7	3.4 Gb
SRX016369	MAQC UHR exp 3 library prep S3	UHR	35 nucleotides	4	1.7 Gb
SRX016370	MAQC UHR exp 3 library prep S4	UHR	35 nucleotides	3	1.6 Gb
SRX016371	MAQC UHR exp 3 library prep S5	UHR	35 nucleotides	4	1.8 Gb
SRX016372	MAQC UHR exp 3 library prep S6	UHR	35 nucleotides	3	1.7 Gb
SRX080222	GSM747473: human_maqc-brain1	HBR	76 nucleotides	1	697.3 Mb
SRX080223	GSM747474: human_maqc-brain2	HBR	76 nucleotides	1	669.5 Mb
SRX080224	GSM747475: human_maqc-UHR1	UHR	76 nucleotides	1	676.7 Mb
SRX080225	GSM747476: human_maqc-UHR2	UHR	76 nucleotides	1	659.9 Mb

### Simulated RNA-Seq data

We simulated 5-reads data for brain tissue and UHR separately (data from a total of 10 reads), which consisted of 20 million single-end reads with quality scores each using the RSEM simulator. We derived model parameters when calculating RSEM expression levels from SRX080222 for human brain simulated data, and from SRX080224 for human UHR simulated data. The parameters used in the simulation are given in the following command.

./rsem-simulate-reads REF/hg uhr_RSEM.stat/uhr_RSEM.model uhr_RSEM.genes.results 0.2 20000000 uhr_read

### MAQC TaqMan qRT-PCR data

MAQC TaqMan qRT-PCR is the benchmark for detecting and quantifying RNA targets [28]. It can be downloaded from the Gene Expression Omnibus with GSE5350 on platform GPL4097 (GEO; http://www.ncbi.nlm.nih.gov/geo/) [29], and comprises 1044 genes from two samples (HBR and UHR). We matched the GEO genes with the UCSC genes and removed gene IDs with duplicate gene names in qRT-PCR. Ultimately, 996 genes were selected for evaluation. For accessions SRX016359, SRX016366, SRX080222, and SRX080223, we used qRT-PCR values of HBR samples, and for the other eight accessions, we used qRT-PCR values of UHR samples.

### Alignment tool

Bowtie is widely used in genome sequencing studies for aligning short DNA sequence reads to large genomes because of its fast and memory-efficient alignments [30]. Bowtie version 0.12.9 was used here to map RNA-Seq reads to regions in the genome. After building an index using human reference transcript data, alignments were performed allowing two mismatches in . SAM format. We allowed multiply mapped reads. We then removed genes with no alignment for accurate evaluation of the normalization methods. The output file was arranged by gene IDs, and because we wanted accurate alignment, we considered any splice-aware mapper.

### Normalization methods

Eight non-abundance and two abundance estimation normalization methods were evaluated in this paper, and calculations were performed on each gene. We defined the gene count of one gene as all the mapped reads of each run.

RC, UQ, and Med use simple descriptive statistical methods that were also widely used in other areas for capturing data characteristics. TMM, DESeq, and Q are proposed for differential gene expression. They are inter-sample normalization methods that give a scaling factor, which scales sample size for each sample. Here, we treated runs in one accession as samples. RPKM does not just consider sample size, but also read length. Here, we treated one run in one accession as one sample. Therefore, for real data with a length of 76 nucleotides, because they only have one run, only RC, RPKM, and ERPKM were applied. A detailed description of each method is as follows.

RC: The raw count for each gene was the sum of gene counts of all runs.

UQ: The upper quartile was calculated by applying the upper quartile of 0.75 to the gene counts of all runs.

Med: The median was calculated as the median of gene counts of all runs.

TMM: The trimmed mean of M-values is a scaling normalization method proposed for differential expression analysis of RNA-Seq data [19]. This normalization method was implemented within the edgeR Bioconductor package. Scaling factors were calculated using the calcNormFactors function in the package, and then rescaled gene counts were obtained by dividing gene counts by each scaling factor for each run. TMM is the sum of rescaled gene counts of all runs.

DESeq: DESeq is a differential gene expression analysis method based on a negative binomial distribution model, with variance and mean linked by local regression, and presents an implementation that also gives scale factors [6]. It is within the DESeq Bioconductor package, and with the estimateSizeFactorsForMatrix function, scaling factors can be calculated for each run. After dividing gene counts by each scaling factor, DESeq values were calculated as the total of rescaled gene counts of all runs.

Q: Quantiles has been previously used to normalize single channel or A-value microarray intensities between arrays [20]. The NormalizeQuantiles function in the Bioconductor package limma [31] normalizes the columns of a matrix to have the same quantiles. Here, we set the total value of function output as the normalization value of the quantiles.

RPKM: This approach quantifies gene expression from RNA-Seq data by normalizing for the total transcript length and the number of sequencing reads. RPKM values can easily be calculated using the definition:

1 $$ \mathrm{RPKM}={10}^9\frac{\mathrm{reads}\ \mathrm{mapping}\ \mathrm{t}\mathrm{o}\ \mathrm{t}\mathrm{ranscript}}{\mathrm{total}\ \mathrm{reads}*\mathrm{transcript}\ \mathrm{length}} $$

ERPKM: Since reads have a non-zero length, and the read probabilities depend on an effective length [32], we calculated the effective reads per kilobase per million mapped reads (ERPKM) using an effective transcript length:

2 $$ \mathrm{effective}\ \mathrm{transcript}\ \mathrm{length} = \mathrm{transcript}\ \mathrm{length} - \mathrm{read}\ \mathrm{length} + 1 $$

Thus, $$ \mathrm{ERPKM}={10}^9\frac{\mathrm{reads}\ \mathrm{mapping}\ \mathrm{t}\mathrm{o}\ \mathrm{t}\mathrm{ranscript}}{\mathrm{total}\ \mathrm{reads}*\mathrm{effective}\ \mathrm{t}\mathrm{ranscript}\ \mathrm{length}} $$(3)

All calculation of non-abundance estimation normalization methods was carried out using R language. R code is provided in Additional file 3.

RSEM and Sailfish are abundance estimation normalization methods. They estimate read count by machine learning methods.

RSEM: RSEM is different from previous normalization methods. It proposes a directed graph model combined with an expectation–maximization algorithm to estimate abundances. RSEM provides a software package for quantifying gene abundances from RNA-Seq data, so we generate a reference index by preparing reference transcript data and calculated RSEM values by inputting RNA-Seq data. The following are the two steps including parameters used in calculating RSEM values:

1. Prepare reference

./rsem-prepare-reference-no-polyA-no-bowtie reference.fa reference_path

2. Calculate RSEM values

./rsem-calculate-expression-sam-p 8 alignmentResults.sam reference_path output_path

Sailfish: Sailfish was introduced to be alignment-free in abundance estimation. It uses the concept of k-mer to index and count RNA-Seq reads. Here, we used the estimated number of k-mers after bias as an estimated count. The following are the two steps including parameters used in computing Sailfish values:

1. Prepare reference

./sailfish index−t reference.fa−o reference_path−k 20

2. Calculate Sailfish values

./sailfish quant−i reference_path−l “T = SE:S = U”−r sequence.fastq−o output_path

Because abundance estimation normalization methods give count estimations for each alignment, RSEM and Sailfish were evaluated by combining with RC and RPKM.

### Statistical analysis

The distribution of qRT-PCR values and all normalization results were tested using the Shapiro-Wilk normality test. According to test results with *p*-values < 0.05, both qRT-PCR values and normalization results were not normally distributed. For the characterization of data, we used Spearman’s rank correlation coefficients to evaluate performance by calculating the similarity between RNA-Seq abundance predictions of each normalization method and the measured qRT-PCR values.

The Spearman correlation coefficient is widely used because it measures linear dependence between two variables as a non-parameter method [33]. It is calculated as the Pearson’s correlation coefficient on the ranks of the data. For a group of genes of size n and the corresponding n raw data, variable X is the qRT-PCR gene expression value, variable Y is the result of the normalization method, and the correlation r_s_ is calculated as:

4 $$ {\mathrm{r}}_{\mathrm{s}}=1-\frac{6{\displaystyle \sum }{\mathrm{d}}_{\mathrm{i}}^2}{\mathrm{n}\left({\mathrm{n}}^2-1\right)} $$

where ${\displaystyle \sum }{\mathsf{d}}_{\mathsf{i}}^{\mathsf{2}} = {\displaystyle \sum_{\mathsf{i}=\mathsf{1}}^{\mathsf{n}}}\ {\left[\mathsf{R}\left({\mathsf{x}}_{\mathsf{i}}\right)\hbox{-} \mathsf{R}\left({\mathsf{y}}_{\mathsf{i}}\right)\right]}^{\mathsf{2}}$ (5), R(x_i_) is the rank of the ith observed value of X, and R(y_i_) is the rank of the ith observed value of Y.

The Spearman correlation coefficient will generate a value between +1 and −1, where +1 indicates a total positive correlation, 0 indicates no correlation, and −1 indicates a total negative correlation. Values closest to +1 or −1 indicate the highest correlation and, therefore, the best normalization results. In this study, the Spearman correlation coefficient was executed by R using the cor.test function.

## **Additional files**

Additional file 1:
**Detailed alignment results for each accession.** (DOCX 25 kb)
Additional file 2:
**Detailed spearman correlation coefficient results for all normalization methods.** (XLSX 17 kb)
Additional file 3:
**R code used for calculating values for each normalization method. **(DOCX 227 kb)
