# Supplementary material for: Comparing the normalization methods for the differential analysis of Illumina high-throughput RNA-Seq data
Source: BMC Bioinformatics. 2015 Oct 28;16:347. doi: 10.1186/s12859-015-0778-7 (PMC4625728; doi:10.1186/s12859-015-0778-7)
Supplement: Additional file 1: — Detailed alignment results for each accession. (DOCX 25 kb) [file 12859_2015_778_MOESM1_ESM.docx]

1. **Alignment results for each accession.**

| **Accession** | **Gene number** | **Run** | **Total read** | **Reads with at least one alignment (with-no-poly-A)** | **Total alignment** |
| --- | --- | --- | --- | --- | --- |
| **SRX016359** | 964 | SRR035678 | 11,712,885 | 535,265 (4.57%) | 4,373,013 |
|  |  | SRR037439 | 11,413,794 | 538,437 (4.72%) | 2,028,240 |
|  |  | SRR037440 | 11,816,021 | 620,617 (5.25%) | 4,385,598 |
|  |  | SRR037441 | 11,244,980 | 566,289 (5.04%) | 2,161,858 |
|  |  | SRR037442 | 12,081,324 | 649,916 (5.38%) | 4,840,368 |
|  |  | SRR037443 | 11,365,146 | 556,873 (4.90%) | 2,043,229 |
|  |  | SRR037444 | 11,616,331 | 510,491 (4.39%) | 1,939,846 |
| **SRX016366** | 964 | SRR037452 | 1,1712,885 | 574,766 (4.91%) | 4,437,550 |
|  |  | SRR037453 | 1,1413,794 | 546,693 (4.79%) | 2,037,851 |
|  |  | SRR037454 | 1,1816,021 | 635,422 (5.38%) | 4,378,263 |
|  |  | SRR037455 | 1,1244,980 | 571,607 (5.08%) | 2,159,821 |
|  |  | SRR037456 | 1,2081,324 | 655,971 (5.43%) | 4,795,036 |
|  |  | SRR037457 | 1,1365,146 | 566,913 (4.99%) | 2,068,179 |
|  |  | SRR037458 | 1,1616,331 | 529,558 (4.56%) | 1,993,062 |
| **SRX016367** | 971 | SRR037445 | 1,2822,531 | 628,142 (4.90%) | 2,129,372 |
|  |  | SRR037446 | 1,3547,513 | 621,698 (4.59%) | 2,094,777 |
|  |  | SRR037447 | 1,3462,723 | 692,086 (5.14%) | 2,348,978 |
|  |  | SRR037448 | 1,3680,634 | 686,959 (5.02%) | 2,550,690 |
|  |  | SRR037449 | 1,3116,688 | 675,199 (5.15%) | 2,328,883 |
|  |  | SRR037450 | 1,3664,929 | 661,282 (4.84%) | 2,711,959 |
|  |  | SRR037451 | 1,2229,347 | 630,531 (5.16%) | 2,268,579 |
| **SRX016368** | 971 | SRR037459 | 1,2822,531 | 633,304 (4.94%) | 2,121,688 |
|  |  | SRR037460 | 1,3547,513 | 661,165 (4.88%) | 2,217,531 |
|  |  | SRR037461 | 1,3462,723 | 704,510 (5.23%) | 2,375,387 |
|  |  | SRR037462 | 1,3680,634 | 698,168 (5.10%) | 2,550,477 |
|  |  | SRR037463 | 1,3116,688 | 687,971 (5.25%) | 2,350,300 |
|  |  | SRR037464 | 1,3664,929 | 679,229 (4.97%) | 2,733,727 |
|  |  | SRR037465 | 1,2229,347 | 638,147 (5.22%) | 2,249,049 |
| **SRX016369** | 950 | SRR037466 | 1,1381,315 | 538,076 (4.73%) | 1,517,367 |
|  |  | SRR037467 | 1,1920,298 | 626,849 (5.26%) | 1,753,141 |
|  |  | SRR037468 | 1,1786,741 | 626,463 (5.31%) | 1,776,096 |
|  |  | SRR037469 | 1,1217,015 | 579,035 (5.16%) | 1,644,307 |
| **SRX016370** | 958 | SRR037470 | 1,4267,937 | 695,300 (4.87%) | 1,863,305 |
|  |  | SRR037471 | 1,4032,875 | 713,469 (5.08%) | 1,943,175 |
|  |  | SRR037472 | 1,3808,725 | 687,773 (4.98%) | 1,861,114 |
| **SRX016371** | 956 | SRR037473 | 1,1776,950 | 525,156 (4.46%) | 1,456,539 |
|  |  | SRR037474 | 1,2323,546 | 650,972 (5.28%) | 1,847,025 |
|  |  | SRR037475 | 1,1773,151 | 624,187 (5.30%) | 1,784,896 |
|  |  | SRR037476 | 1,2135,562 | 611,637 (5.04%) | 1,719,499 |
| **SRX016372** | 962 | SRR037477 | 1,6066,286 | 761,677 (4.74%) | 1,985,437 |
|  |  | SRR037478 | 1,5720,055 | 753,105 (4.79%) | 1,959,320 |
|  |  | SRR037479 | 1,5587,049 | 747,093 (4.79%) | 1,963,831 |
| **SRX080222** | 817 | SRR299109 | 15262240 | 322193 (2.11%) | 322824 |
| **SRX080223** | 813 | SRR299110 | 14871450 | 311240 (2.09%) | 311899 |
| **SRX080224** | 828 | SRR299111 | 15077912 | 469007 (3.11%) | 469690 |
| **SRX080225** | 830 | SRR299112 | 14888906 | 443845 (2.98%) | 444486 |
| **Simulated-Brain** | 787 | Brain1 | 20000000 | 478955 (2.39%) | 656590 |
|  |  | Brain2 | 20000000 | 477763 (2.39%) | 653319 |
|  |  | Brain3 | 20000000 | 477550 (2.39%) | 653524 |
|  |  | Brain4 | 20000000 | 477783 (2.39%) | 654727 |
|  |  | Brain5 | 20000000 | 479511 (2.40%) | 655104 |
| **Simulated-UHR** | 819 | UHR1 | 20000000 | 703029 (3.52%) | 975175 |
|  |  | UHR2 | 20000000 | 707647 (3.54%) | 979813 |
|  |  | UHR3 | 20000000 | 704835 (3.52%) | 976265 |
|  |  | UHR4 | 20000000 | 706956 (3.53%) | 978831 |
|  |  | UHR5 | 20000000 | 706027 (3.53%) | 976932 |

1. **Number of genes with different alignment counts on accession SRA010153.1, which shows the breakdown of the number of genes containing various ranges of alignment count.**

| **Alignment** | **>1,000,000** | **>100,000** | **>10,000** | **>1,000** | **>100** | **>10** | **>0** |
| --- | --- | --- | --- | --- | --- | --- | --- |
| **Gene number** | 14 | 152 | 524 | 837 | 933 | 972 | 996 |
