# Supplementary material for: Comparing the normalization methods for the differential analysis of Illumina high-throughput RNA-Seq data
Source: BMC Bioinformatics. 2015 Oct 28;16:347. doi: 10.1186/s12859-015-0778-7 (PMC4625728; doi:10.1186/s12859-015-0778-7)

1. **R code used in normal distribution test**

> rawData <- read.table(file.path("input.txt"), header=TRUE)

> x <- rawData[,c(1)]

> shapiro.test(x)

1. **R code used in calculating Spearman correlation**

inputFile <- "data/uhr.txt"

rawData <- read.table(file.path(inputFile), header=TRUE, sep="\t")

x <- rawData[,c(1)]

a1 <- rawData[,c(2)]

cor.test(x,a1,method="spearman")

1. **R code used in calculating eight non-abundance estimation methods**

library(limma)

library(edgeR)

library(DESeq)

inputFile <- " input.txt"

rawData <- read.table(file.path(inputFile), header=TRUE, sep="\t")

geneLength <- rawData[,c(2)]

geneLengthE <- rawData[,c(3)]

geneCount <- rawData[,c(5:11)]

RC <- rowSums(geneCount)

UQ <- apply(geneCount, 1, quantile, 0.75)

Med <- apply(geneCount, 1, median)

f <- calcNormFactors(geneCount, method="TMM")

TMM <- rowSums(geneCount / f)

ef <- estimateSizeFactorsForMatrix(geneCount)

DESeq <- rowSums(geneCount / ef)

MQ <- limma::normalizeQuantiles(geneCount)

Q <- rowSums(MQ)

lengthsInKb <- geneLength/1000

millionMapped <- sum(RC)/1e+06

rpm <- RC/millionMapped

RPKM <- rpm/lengthsInKb

lengthsEInKb <- geneLengthE/1000

ERPKM <- rpm/lengthsEInKb

geneCountNorm <- cbind(RC, UQ, Med, TMM, DESeq, Q, RPKM, ERPKM)

write.table(geneCountNorm, file=" NormResults.txt")

Input file example for R code


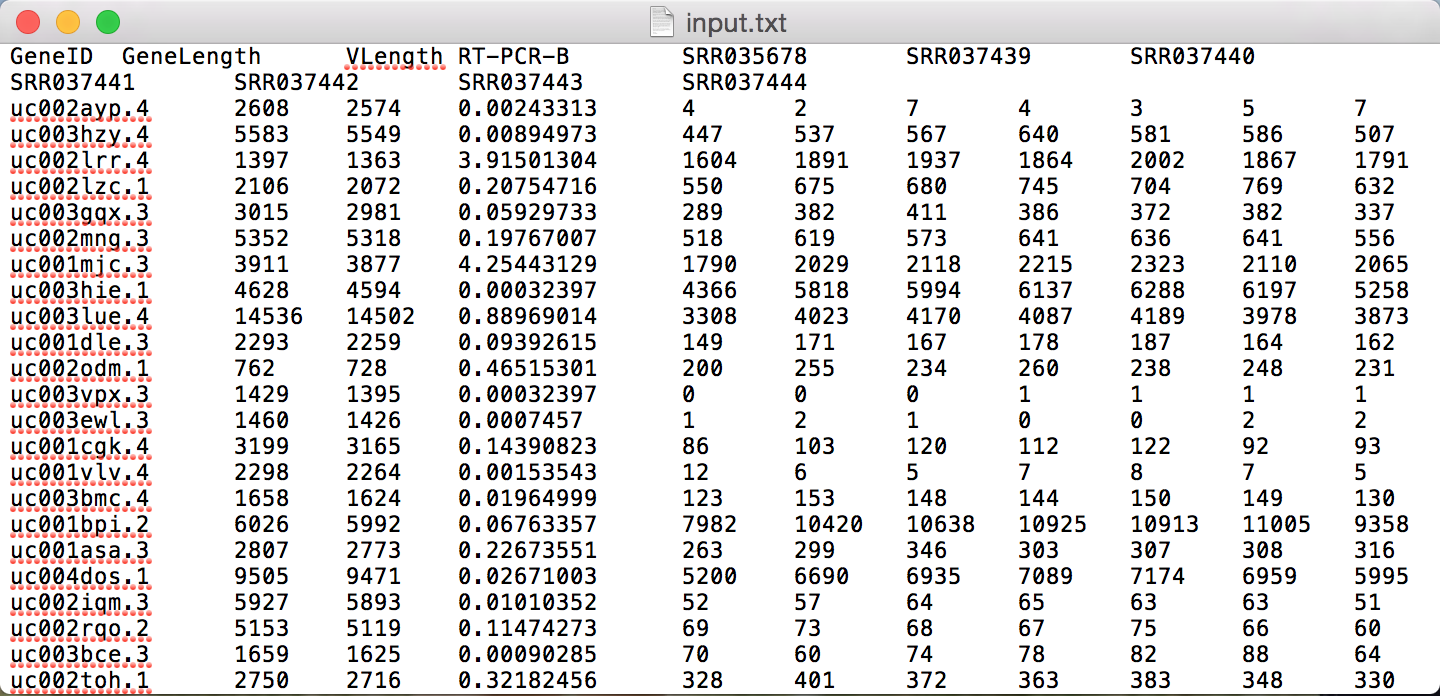

Supplement: Additional file 3: — R code used for calculating values for each normalization method. (DOCX 227 kb) [file 12859_2015_778_MOESM3_ESM.docx]
